# Progress of COVID-19 Epidemic in Pakistan

**DOI:** 10.1177/1010539520927259

**Published:** 2020-05-19

**Authors:** Khadijah Abid, Yashfika Abdul Bari, Maryam Younas, Sehar Tahir Javaid, Abira Imran

**Affiliations:** 1 College of Physicians and Surgeons, Karachi, Pakistan; 2 Liaquat National Hospital, Karachi, Sindh, Pakistan

**Keywords:** coronavirus, COVID-19, public health, virus outbreak, pandemic, epidemic, coronavirus, epidemiology, population health, Pakistan

## Abstract

The outbreak of corona virus initiated as pneumonia of unknown cause in December 2019 in Wuhan, China, which has been now spreading rapidly out of Wuhan to other countries. On January 30, 2020, the World Health Organization (WHO) declared coronavirus outbreak as the sixth public health emergency of international concern (PHEIC), and on March 11, 2020, the WHO announced coronavirus as *pandemic*. Coronavirus is thought to be increasing in Pakistan. The first case of coronavirus was reported from Karachi on February 26, 2020, with estimated populace of Pakistan as 204.65 million. Successively, the virus spreads into various regions nationwide and has currently become an epidemic. The WHO has warned Pakistan that the country could encounter great challenge against the outbreak of coronavirus in the coming days. This short communication is conducted to shed light on the epidemic of coronavirus in the country. It would aid in emphasizing the up-to-date situation in a nutshell and the measures taken by the health sector of Pakistan to abate the risk of communication.

## Background

The outbreak of coronavirus initiated as pneumonia of unknown cause in December 2019 in Wuhan, China, which has been now spreading rapidly out of Wuhan to other countries.^1^ On January 30, 2020, the World Health Organization (WHO) declared COVID-19 outbreak as the sixth public health emergency of international concern (PHEIC), and on March 11, 2020, the WHO announced COVID-19 as *pandemic*.^2^ On April 9, 2020, nearly 1 436 198 cases of 2019-novel coronavirus recorded out of which 85 522 died with a case fatality rate (CFR) of 5.95%. The WHO evaluated the global risk of COVID-19 as very high. In the coming days and weeks, the amount of events, fatalities, and affected countries is projected to increase sharply.^1^

COVID-19 is thought to be expanding in Pakistan. The first case of COVID-19 was reported from Karachi on February 26, 2020, with estimated populace of Pakistan as 204.65 million.^3,4^ Successively, the virus spreads into various regions nationwide and has currently become epidemic. Within 45 days, on April 10, 2020, the Pakistan’s tally has reached 4601 confirmed cases of COVID-19, 727 patients have recovered, and 66 have died.^4^

This short communication is conducted to shed light on the epidemic of coronavirus in the country. It would aid in emphasizing the up-to-date situation in a nutshell and the measures taken by the health sector of Pakistan to abate the risk of communication.

## Situational and Epidemiological Analysis

**Figure 1.**
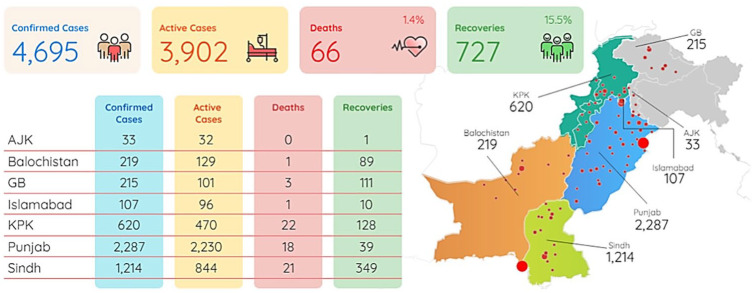
Pakistan situation report (April 10, 2020—7:20 pm).

According to Pakistan’s last update^4^ at 9:17 am on April 10, 2020, 54 706 suspected coronavirus cases were reported in Pakistan, 4695 of which tested positive for COVID-19 (8.6%). Of the 4695 cases, 727 patients recovered (15.5%), 45 remain critical (1%), and 66 died (CFR: 1.4%). Coronavirus attack rate is estimated to be 2.3 per 100 000 Pakistani population. Nearly 49% of cases are registered from Punjab. The cumulative reported cases of COVID in Punjab increased by 53.2% (1493 cases to 2287 cases) from April 5 to April 10, 2020. Sindh recorded the second highest number of incidences (26%) followed by Khyber Pakhtunkhwa (KPK; 13.2%). From April 5 to April 10, 2020, the cumulative reported cases of COVID increased by 28% (881 cases to 1128 cases) in Sindh and in KPK from 205 cases to 620 cases. Azad Jammu Kashmir (AJK) recorded the lowest number of incidences (0.7%) followed by Islamabad (2.3%). District wise, 880 cases have been confirmed from Lahore (19%) and 871 cases from Karachi (18.9%). Gilgit Baltistan (GB) has the highest rate of recovery (52%), while KPK has the lowest rate of mortality (3.5%) compared with other regions of Pakistan (Figure 1).

**Figure 2.**
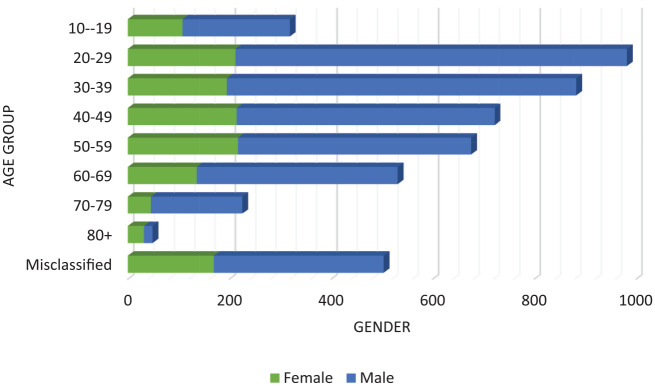
Age and gender-wise distribution of COVID-19.

About 28.2% of the 4695 cases are female, and 71.8% are male. Overall, the 20 to 39 age group is most affected by COVID-19, in which 21.8% being females and 78.2% being males. In Baluchistan, most cases belonged from age group 22 to 48 years, in Sindh from age group 22 to 52 years, and in Punjab from age group 22 to 44 years. Whereas in AJK and GB age varies from 31 to 60 years. When planning initiatives, attention must be given to the age groups mentioned above^4^ (Figure 2).

About 138 health care professionals have contracted coronavirus. Major chunk of infected health care professionals were from Sindh (61.5%), 18.1% were from Baluchistan, 8.7% were from Punjab, and 1% to 5% were from KPK, Islamabad, AJK, and GB. Out of 138 health care professionals, 48% were from age group 21 to 40 years, followed by 40% from age group 41 and older. Majority of the infected health care professionals are male.^5^

## Discussion

Recently, the incidence of COVID-19 has increased due to travel to other parts of the world. Pakistan has trade and travel with Iran and China. The increased influx of travelers through land, air, and sea put Pakistan at higher odds of further spread of coronavirus from neighboring countries. The possibility of further importation of the virus into Pakistan is very high, and because Pakistan has already imported the virus, the country needs to take stringent measures to detect potential cases early in order to curb existing epidemic and tracking steps to prevent further spread. After the unexpected rise in coronavirus cases in Pakistan, the Government of Pakistan has halted trade and transport operations with Iran. Now owing to severe snowfall the land connection with China is blocked. Whereas mobilization is tightly controlled at the boundaries of Chaman and Taftan. Additionally, it monitors external travel to Iraq, the Kingdom of Saudi Arabia, and Iran. Weekly, 41 flights operate in Pakistan from three cities (Karachi, Islamabad, and Lahore) and to two destinations in China (Urumqi and Beijing). The danger of virus importation into Pakistan is very high and needs good precautions and strict steps to identify possible cases early and surveillance steps to deter further virus transmission. Pakistan is still in the containment process. The step to shutdown point of entry and borders has been taken. All international flights have been cancelled. In this way, the number of cases might not increase as most of the spread was through worldwide migrants and yet it is impossible to find the point of origin.

With increasing cases of immensely contagious COVID-19, Pakistan’s economy is under great deterioration. The terror of fatal disease and economic distress have come up together. The country cannot bear extended lockdown and should the lockdown extend, Pakistan will suffer unmanageable economic loss. Pakistan does not have any sufficient resources to provide for the patients at the moment. Most of the populace is working on daily wages. The shutdown of the whole country would cause death either due to hunger or from COVID-19. The current statement of Pakistan’s prime minister calls for a community meeting among susceptible countries that are dealing with the pandemic. It has been decided that rather than complete shutdown, people should avoid mass gatherings, and partial shutting down of the country will take place in order for the economy to provide for basic necessities.^4^

## Conclusion and Recommendations

COVID-19 is swiftly spreading worldwide. Within a few months, the mortality rate and morbidity rate has reached unexpected levels. The clinicians are working to invent treatments and vaccine to prevent this infection. The extreme situation is yet to occur. However, if we take one step toward self-isolation, it could save the entire community and the risk will decline immediately. This is a situation where each individual has to take steps toward minimizing the risk by staying in the house and immobilizing themselves. The airborne, contact transmission can only be disinfected if proper handwashing protocols are followed and each individual carry out precautionary measures to safe other individuals from this debilitating virus. Pakistan has a tremendous potential in public health, and different sectors can work together to address the challenges by the engagement of society and community along with policy initiatives.
